# Higher Sensitivity of Xpert MTB/RIF Ultra Over Tuberculosis Culture for the Diagnosis of Spinal Tuberculosis With Open or Computed Tomography–Guided Biopsies

**DOI:** 10.1093/ofid/ofad621

**Published:** 2023-12-07

**Authors:** Robyn Waters, Maritz Laubscher, Robert N Dunn, Nawaal Adikary, Anna K Coussens, Michael Held

**Affiliations:** Orthopaedic Research Unit, Division of Orthopaedic Surgery, Groote Schuur Hospital, Cape Town, South Africa; Division of Orthopaedic Surgery, Groote Schuur Hospital, Cape Town, South Africa; Centre for Infectious Disease Research in Africa, Institute of Infectious Disease and Molecular Medicine, Faculty of Health Sciences, University of Cape Town, Cape Town, South Africa; Orthopaedic Research Unit, Division of Orthopaedic Surgery, Groote Schuur Hospital, Cape Town, South Africa; Division of Orthopaedic Surgery, Groote Schuur Hospital, Cape Town, South Africa; Orthopaedic Research Unit, Division of Orthopaedic Surgery, Groote Schuur Hospital, Cape Town, South Africa; Division of Orthopaedic Surgery, Groote Schuur Hospital, Cape Town, South Africa; Microbiology Diagnostic Laboratory, National Health Laboratory Service, Groote Schuur Hospital, Cape Town, South Africa; Centre for Infectious Disease Research in Africa, Institute of Infectious Disease and Molecular Medicine, Faculty of Health Sciences, University of Cape Town, Cape Town, South Africa; Infectious Diseases and Immune Defence Division, Walter and Eliza Hall Institute of Medical Research, Parkville, Australia; Department of Medical Biology, University of Melbourne, Parkville, Australia; Orthopaedic Research Unit, Division of Orthopaedic Surgery, Groote Schuur Hospital, Cape Town, South Africa; Division of Orthopaedic Surgery, Groote Schuur Hospital, Cape Town, South Africa

**Keywords:** diagnosis, *Mycobacterium tuberculosis*, spinal tuberculosis, tuberculosis, tuberculosis diagnosis

## Abstract

**Background:**

Diagnostic specimens for spinal tuberculosis (STB) are mostly collected via open surgery. Percutaneous computed tomography (CT)–guided biopsies are used in times of limited surgical availability. However, poor diagnostic accuracy of *Mycobacterium tuberculosis* (*Mtb*) culture has been reported with this method, due to limited sample volume and the paucibacillary nature of STB. We evaluated Xpert MTB/RIF Ultra on open and CT-guided biopsies as compared with the gold standard *Mtb* culture and histopathology.

**Methods:**

We conducted a prospective diagnostic accuracy study of Xpert Ultra, as compared with tuberculosis culture and histopathology, in adults with signs and symptoms of STB at a tertiary academic hospital in South Africa from November 2020 to December 2021. Diagnostic testing was performed on 31 patients with available samples.

**Results:**

Xpert Ultra had a sensitivity of 94.7% (95% CI, 75.3%–99.7%) and specificity of 100% (95% CI, 75.7%–100.0%) against a reference standard of *Mtb* culture and histopathology. Xpert Ultra had high diagnostic accuracy in open and CT-guided biopsy samples with sensitivity and specificity of 100% and 100% (open) and 89% and 100% (CT), respectively. *Mtb* culture had limited specificity for CT-guided biopsies (43%; 95% CI, 15.8%–74.9%). HIV-1 coinfection did not affect *Mtb* abundance measures by Xpert Ultra or culture. Xpert Ultra was also superior to culture for STB diagnosis in patients concurrently treated for pulmonary tuberculosis.

**Conclusions:**

Xpert Ultra detected more STB cases than culture for CT-guided biopsy samples. There was also no difference in sensitivity for open biopsies, irrespective of HIV-1 status, making it an important tool for rapid diagnosis, especially during times or in locations where open surgery is not possible or concurrent pulmonary tuberculosis treatment is initiated.

## Teaser

Xpert MTB/RIF Ultra has a high diagnostic accuracy for spinal tuberculosis in both open and CT-guided biopsy samples, irrespective of HIV-1 coinfection, while tuberculosis culture has limited sensitivity when using CT-guided biopsies, particularly in patients on concurrent pulmonary tuberculosis treatment.

Spinal tuberculosis (STB) is a debilitating form of extrapulmonary tuberculosis (TB) accounting for up to 50% of all osteoarticular TB cases [1–6]. Tissue-based diagnosis of *Mycobacterium tuberculosis* (*Mtb*) infection and rapid appropriate initiation of antituberculous therapy are required to prevent serious deformity, neurologic complications, disability, and ongoing health care expenses [7]. Although antibiotic management is the mainstay of treatment, surgery is indicated for mechanical stabilization or decompression of vertebral column destruction. For patients treated nonsurgically, a reliable confirmation of TB diagnosis is crucial, especially in populations with high resistance to certain TB drugs [7, 8].

The gold standard method for bacteriological confirmation of *Mtb* is liquid culture; however, this is not used as a primary diagnostic test in many high-burden countries due to cost, the biological safety level laboratory infrastructure required, and the long lead times to positive results [9]. Furthermore, coinfection with HIV-1 is frequent, often leading to paucibacillary specimens and limited sensitivity [10]. The design and implementation of improved nucleic acid amplification–based tests, such as Xpert MTB/RIF Ultra (Xpert Ultra), have been a major advance in TB diagnosis [11–17], especially in paucibacillary extrapulmonary TB [18–22]. Xpert Ultra has shown improved sensitivity as compared with the original Xpert MTB/RIF (Xpert) in detecting *Mtb*, with a limit of detection of 15 bacterial colony-forming units/mL in sputum and enhanced rifampicin (RIF) resistance detection [11, 23]. Therefore, the World Health Organization recommends its use to diagnose extrapulmonary TB over conventional culture and phenotypic drug sensitivity testing [17].

A meta-analysis of 19 studies investigating osteoarticular TB [21] revealed that Xpert MTB/RIF has a pooled sensitivity and specificity of 81% (95% CI, 77%–84%) and 99% (95% CI, 97%–100%), respectively, as compared with a composite reference standard for all sample types. When analysis was restricted to tissue samples, a sensitivity of 84% (95% CI, 76%–90%) and a specificity of 98% (95% CI, 94%–99%) were reported [21]. When compared with culture alone, Xpert had a sensitivity of 96% (95% CI, 90%–98%) and a specificity of 85% (95% CI, 57%–96%). A recent study found higher sensitivity for Xpert Ultra (90.91%) than for Xpert (78.79%) and culture (39.39%) in pus samples from various musculoskeletal sites, including the spine [20]. So far, no data on the diagnostic accuracy of Xpert Ultra have been reported for open or closed tissue biopsy in STB.

During the COVID-19 pandemic, patient presentation was heavily affected by reduced hospital staff capacity and access to operating theaters [24]. Groote Schuur Hospital is a tertiary-level hospital in the Western Cape province of South Africa that admits around 80 patients with suspected spinal TB annually [25–28]. During the first 2 years of the COVID-19 pandemic, patients who did not require open surgery underwent a percutaneous computed tomography (CT)–guided biopsy, where a considerably reduced volume of tissue was collected, thereby potentially decreasing the diagnostic sensitivity. The aim of this study was to explore the use of Xpert Ultra, as compared with liquid TB culture, in the detection of *Mtb* in open and percutaneous CT-guided biopsy samples.

## METHODS

### Study Design

Patients for this prospective diagnostic accuracy study were recruited between November 2020 and December 2021. All patients were recruited via the Division of Orthopaedic Surgery within Groote Schuur Hospital, a public hospital servicing a population of low socioeconomic backgrounds and low-income households in densely populated vulnerable communities.

### Patient Consent Statement

Written consent was obtained from adults (age >18 years) with signs and symptoms of STB who were consecutively enrolled. Clinical and radiologic red flags were included. Clinical red flags were back pain, constitutional symptoms (eg, cough, fever, weight loss), immune compromise (eg, HIV-1 infection, diabetes mellitus), neurologic deficit, elevated C-reactive protein and erythrocyte sedimentation rate, and local kyphosis [29]. Radiologic red flags were paraspinal/vertebral shadow, vertebral body destruction, loss of anterior vertebral height, adjacent vertebral endplate changes with preserved disc height, presence of cold abscess, and/or cord compression [7, 29]. Ethical and institutional approval was received from the University of Cape Town Faculty of Heath Sciences (HREC REF 606/2019).

### Data Collection

Demographic data, initial clinical presentation, and laboratory parameters were collected through a combination of interviews, electronic medical and laboratory records, and hospital folders. The following data were also collected: clinical and radiologic red flags, results of histopathology, HIV-1 infection status, SARS-CoV-2 results, CD4 cell count and HIV-1 viral load (VL), pulmonary TB (PTB) history, Xpert Ultra cycle threshold (Ct) values (the number of DNA amplification cycles to positive *Mtb* detection: the lower the Ct value, the higher the *Mtb* burden) for *Mtb* genes *rpoB* and *IS1081-IS6110*, and TB mycobacteria growth indicator tube (MGIT) culture result and time to positivity (TTP). All data were entered into a study-specific REDCap database hosted by University of Cape Town [30, 31].

### Diagnostic Sampling and Histopathology

Biopsies were collected under strict aseptic conditions in the operating theater and taken from radiographically predetermined areas of spinal disease, such as granuloma/abscess and vertebral destruction. Indications for open surgery included instability of the spinal column, acute deterioration of neurology, and a large paravertebral abscess. Abscesses were drained through a costotransversectomy in the thoracic spine and through an anterolateral retroperitoneal approach in the lumbar spine. A thoracotomy was done when stabilization of the anterior column of the thoracic spine was necessary. The Smith-Robinson [32] approach was used for cervical spine lesions. Biopsy material included tissue biopsies from diseased bone or soft tissue. Percutaneous CT-guided biopsies, ranging from 2 to 7 mm long by 1 mm wide, were reserved for patients who did not require drainage or stabilization. A percutaneous transpedicular biopsy was performed in the thoracic spine and a percutaneous paraspinal biopsy in the lumbar spine. A single tissue sample was submitted for diagnostic testing to limit discrepancies, reduce the risk of error, and maximize the specimen area for possible TB isolation. Samples were either submitted on the same day immediately postsurgery or stored at 4 °C and submitted the following morning, if surgery was conducted during the night. An approximate 1-cm^2^ section of this tissue was subjected to Xpert Ultra and MGIT TB culture. Histology was performed per hospital guidelines. Necrotizing granulomatous inflammation was indicative of STB. Direct microscopy for *Mtb* was performed with a Ziehl-Neelsen stain for microbiology and hematoxylin-eosin stain for histopathology [29, 33].

### Xpert Ultra Testing on Spinal Biopsy

Xpert Ultra was performed by a trained medical technologist at the National Health Laboratory Service per the manufacturer's instructions. Briefly, spine biopsy tissue specimens in saline solution were mechanically homogenized. Approximately 1 cm^2^ of open biopsy tissue or 2 to 7 mm of CT-guided biopsy was placed in a sterile mortar and pestle, to which 1 mL of sterile saline was added and mechanically crushed. Afterward, 500 µL of crushed specimen was mixed with 1.5 mL of sample reagent, vortexed for 10 seconds, and incubated at room temperature for 10 minutes. The mixture was vortexed for another 10 seconds and incubated at room temperature for a further 5 minutes. The 2-mL mixture was transferred into the cartridge and run on the GeneXpert MTB/RIF instrument (Cepheid). When the *Mtb* target is present within the sample, Xpert Ultra provides a semiquantitative result, defined by the manufacturer as follows: trace, very low, low, medium, or high.

### MGIT TB Culture and Drug Susceptibility Testing

The automated BACTEC MGIT 960 (BD Diagnostic Systems) was used for *Mtb* culture and was performed at the National Health Laboratory Service. The remaining 500 µL of homogenized sample in saline was used for MGIT culturing. Drug sensitivity testing was performed in the same laboratory on positive cultures with the Hain MTBDR+ line probe assay.

### TB Definitions

Patients were divided into 3 groups: *definite STB* was defined as TB culture positive and histopathology suggestive of *Mtb* infection. In patients with negative *Mtb* cultures, *probable STB* was diagnosed if histopathologic, clinical, and radiologic findings were suggestive of *Mtb* infection. *Not STB* was defined as culture and histopathology negative. Definite STB and probable STB were defined as true positive when calculating sensitivity and specificity.

### TB Treatment

Study patients with diagnosed drug-sensitive STB were prescribed RIFAFOUR—a combination of RIF, isoniazid, pyrazinamide, and ethambutol—for approximately 9 to 12 months, as established common practice in the Western Cape. In most cases, vitamin B6 (pyridoxine) supplementation was provided to prevent the development of peripheral neuropathy [7, 34]. Patients with PTB were on prior treatment according to South African national TB guidelines.

### Statistical Analysis

All data were analyzed with Prism version 9.3.1 (GraphPad). The sensitivity, specificity, and positive and negative predictive values of Xpert Ultra with 95% CIs were determined. Simple descriptive statistics were used to characterize the cohort. Nonnormally distributed continuous data were summarized by median and IQR. Categorical data were summarized as frequency and percentage. Statistical tests included Mann-Whitney *U* test, chi-square test, and Wilcoxon rank sum test. Differences were considered statistically significant at *P* < .05.

## RESULTS

### Cohort Demographic and Clinical Details

**Figure 1.**
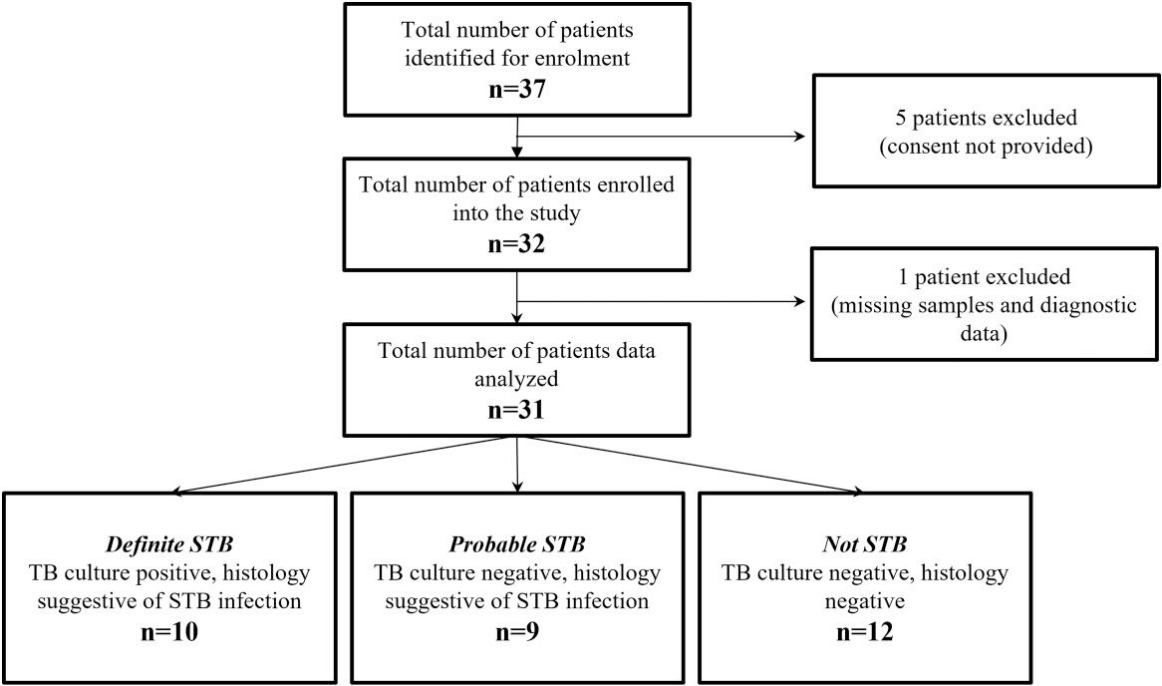
Study flow diagram shows the number of individuals screened, consented, and excluded and the diagnostic definitions. STB, spinal tuberculosis; TB, tuberculosis.

An overall 37 patients with signs and symptoms of STB were identified for study recruitment; 32 consented and were prospectively enrolled; and 31 were included in the final data analysis (Figure 1). Nineteen patients (61%) were STB histopathology positive (acid-fast bacteria positive: n = 3, 16%). Ten (32%) were classified as definite STB with the other 9 (29%) as probable STB and 12 (39%) as not STB (alternate diagnosis reported in Supplementary Table 1).

Of the 31 enrolled, the median age was 47 years (IQR, 37–53), and 14 (45%) were male. Fifteen (48%) were HIV-1 infected, and all reported adherence to antiretroviral therapy (ART). The mean CD4 count in patients who were HIV-1 infected was 319 cells/µL (SD, 207). Of the 13 with VL data (2 missing VL), only 4 (31%) had undetectable VL despite ART adherence. The median VL for patients with detectable and reported VL was 372 copies/mL (IQR, 50–676). Nine (29%) patients were undergoing antituberculous therapy for concurrent PTB, of whom 8 (89%) were HIV-1 coinfected. Fourteen patients had previous PTB (HIV-1 coinfected: n = 10, 71%), of which 13 (93%) had completed first-line antituberculous therapy (Table 1).

**Table 1.** Demographic and Clinical Characteristics of the Overall Cohort and Stratified by STB Diagnostic Group

	STB, No. (%) or Median (IQR)				
Characteristic	Overall	Definite	Probable	Not	*P* Value
Patients	31	10 (31)	9 (29)	12 (39)	
Age, y	47 (37–53)	41 (29–51)	40 (36–44)	55 (48–62)	.**0075**
Male gender	14 (45)	5 (50)	5 (55)	4 (33)	.46
PTB history	14 (45)	5 (50)	4 (44)	5 (33)	>.99
HIV-1					
Positive	15 (48)	6 (60)	6 (67)	3 (25)	.066
Viral load, copies/mL	372 (211–409)	372 (211–409)	594 (42–13 343)	60–676^a^	.9310
CD4 count, cells/µL	312 (173–411)	359 (197–589)	275 (193–416)	225 (140–277)	.5726
Antituberculous therapy before biopsy	10 (32)	3 (30)	6 (67)	1^b^ (8)	.**046**
Mortality	4 (13)	1 (25)	0 (0)	3 (75)	…
Positive^c^					
Histopathology	15/25 (60)	7/7 (100)	8/8 (100)	0/10 (0)	…
Histology AFB	3/25 (12)	2/7 (29)	1/8 (13)	0/10 (0)	…
TB culture					
Positive^d^	10/29 (34)	10 (100)	0/7 (0)	0 (0)	…
TTP, d	10 (32)	16 (12–22)	…	…	…
Xpert					
Ultra positive	18 (58)	10 (100)	8 (89)	0 (0)	…
IS1081-IS6110 Ct, cycles	…	19.3 (16.7–20.8)	20.9 (18.3–23.6)	…	.1318^e^
*rpoB* Ct, cycles	…	26.1 (22.1–28.6)	27.8 (24.8–30.9)	…	.2658^e^

*P* values were calculated with a Mann-Whitney test or Fisher exact test comparing STB (definite and probable combined) vs not STB. Bold indicates *P* < .05. Ellipses (…) indicate no statistical tests employed. Three patients had positive SARS-CoV-2 results: 2 probable STB and 1 not STB.

Abbreviations: AFB, acid-fast bacteria; Ct, cycle threshold; PTB, pulmonary tuberculosis; STB, spinal tuberculosis; TB, tuberculosis; TTP, time to positivity.

^a^Viral load for 2 of 3 patients only.

^b^Patient was initiated on antituberculous therapy 2 days prior to biopsy with evidence of miliary TB.

^c^Histopathology results were available for 25 patients.

^d^TB cultures were performed for 29 patients; 2 patients did not have culture performed due to insufficient sample.

^e^Comparing definite and probable STB.

Patients with definite and probable STB were significantly younger than patients without STB (*P* = .02). There was a trend for higher frequency of HIV-1 coinfection in those with definite or probable STB (60% and 67%, respectively) vs not STB (25%, *P* = .066). Half (5/10) of those with a definite STB diagnosis reported previous TB, as opposed to 33% (5/12) for those without STB, although this was not significantly different. The overall mortality rate was 13% (n = 4).

### High Accuracy of Xpert Ultra for STB Diagnosis

**Figure 2.**
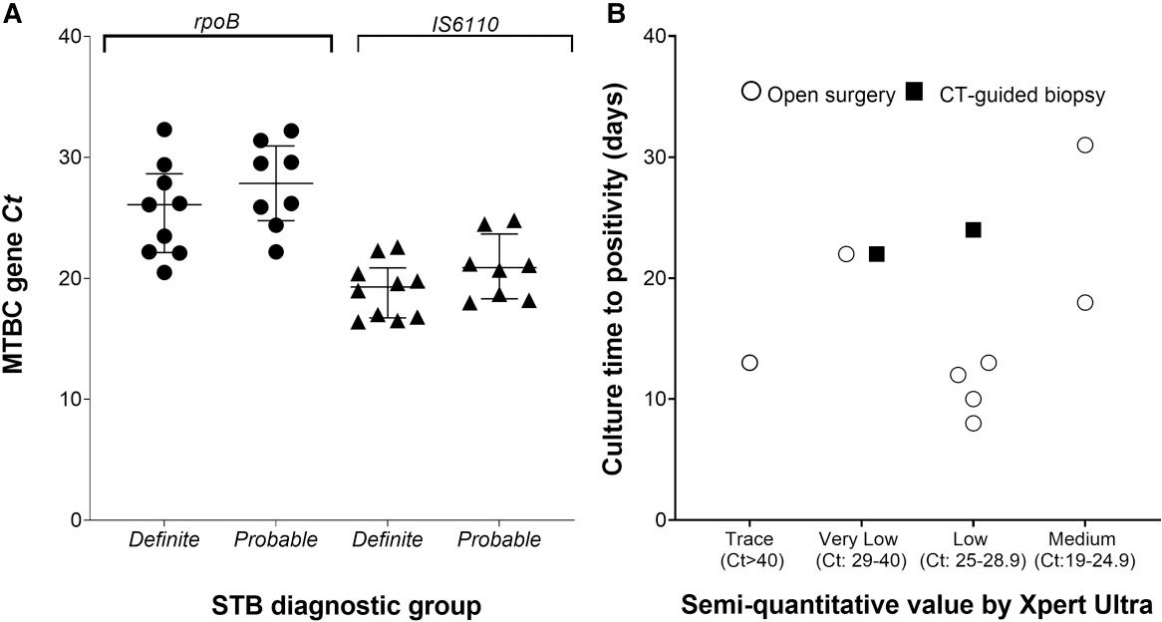
*A*, Difference in MTBC gene Ct values for *rpoB* or *IS1081–IS6110* between definite and probable STB. Data are presented as median (IQR). *B*, Time to positive detection of *Mtb* in culture classified according to the semiquantitative value provided by Xpert Ultra. CT, computed tomography; Ct, cycle threshold; MTBC, *Mycobacterium tuberculosis* complex; STB, spinal tuberculosis.

Xpert Ultra test results were available within 24 to 48 hours as compared with a median culture TTP of 16 days (IQR, 12–22). No RIF resistance was noted. Xpert Ultra confirmed detection of *Mtb* DNA in all 10 (100%) patients with definite STB and further detected *Mtb* DNA in biopsy samples from 8 of 9 (89%) patients with probable STB, including 5 of 6 (83%) from those who were undergoing empiric TB treatment for concomitant PTB prior to biopsy. There was no difference in *Mycobacterium tuberculosis* complex gene Ct values for *rpoB* or *IS1081-IS6110* between definite and probable STB (Table 1, Figure 2*A*). There was also no relationship between the semiquantitative readouts for Xpert Ultra Ct ranges and culture TTP (Figure 2*B*).

For the composite reference standard of any STB, the per-sample sensitivity of Xpert Ultra was 94.7% (95% CI, 75.3%–99.7%) and the specificity was 100% (95% CI, 75.7%–100.0%). A secondary analysis comparing Xpert Ultra with *Mtb* culture–positive samples revealed a sensitivity of 100% (95% CI, 72.3%–100%) and a reduced specificity of 68.4% (95% CI, 46.0%–84.6%) due to Xpert Ultra detecting *Mtb* DNA in culture-negative samples from patients who had initiated treatment prior to biopsy (Table 2).

**Table 2.** Diagnostic Accuracy of Xpert Ultra for Spinal TB Diagnosis

	% (No., 95% CI)			
Reference Standard	Sensitivity	Specificity	PPV %	NPV %
TB positive				
Composite: culture or histology	94.7 (18/19, 75.3–99.7)	100.0 (12/12, 75.7–100.0)	94.7	91.7
Culture only	100.0 (10/10, 72.3–100)	68.4 (13/19, 46.0–84.6)	79.5	100.0

Abbreviations: NPV, negative predictive value; PPV, positive predictive value; TB, tuberculosis.

### Equivalent Accuracy of Xpert Ultra in Open and CT-Guided Biopsy Samples

Next, we compared the diagnostic performance of Xpert Ultra with histopathology and *Mtb* culture between biopsy sample types. There was no difference in age or gender between patients who underwent the different biopsy methods (Table 3), but those who had a CT-guided biopsy were more likely to be HIV-1 positive (73% vs 35%, *P* = .0659) and receiving TB treatment prior to biopsy (64% vs 15%, *P* = .0134). Of the 20 (65%) patients who underwent open biopsies, 10 (50%) were diagnosed with STB. In contrast, of the 11 (35%) patients who underwent percutaneous CT-guided biopsies, 9 (82%) were diagnosed with STB. Xpert Ultra was positive in 8 of 9 (89%) CT-guided biopsies vs only 2 of 9 (22%) by culture. Of the 7 culture-negative samples, 6 were from patients who received TB treatment prior to CT-guided biopsy (Supplementary Table 2). There was no significant difference in Xpert Ultra Ct values or culture TTP between biopsy types, although there was a trend for a lower *rpoB* Ct in CT-guided biopsies (*P* = .0876).

**Table 3.** Demographic and Clinical Characteristics of Cohort by Diagnostic Sampling Method

	Biopsy, No. (%) or Median (IQR)		
	Open (n = 20)	CT Guided (n = 11)	*P* Value
Age, y	48 (38–59)	42 (37–51)	.3637
Male gender	9 (45)	5 (45)	…
Antituberculous therapy prior to biopsy^a^	3 (15)	7 (64)	**.0134**
HIV-1 positive	7 (35)	8 (73)	.0659
Any STB diagnosed	10 (50)	9 (82)	…
Definite: histology positive, culture positive	8/10 (80)	2/9^b^ (22)	…
Probable: histology positive, culture negative/NA	2/10 (20)	7/9^b^ (78)	…
Not: histology negative, culture negative	10 (50)	2 (18)	…
Positive culture TTP, d	13 (11–21)	23 (22–24)	.222
Xpert Ultra positive STB	10/10 (100)	8/9 (89)	…
Ct, cycles			
IS1081-IS6110	19.4 (16.7–21.1)	20.3 (18.3–22.2)	.359
*rpoB*^c^	25.9 (22.1–27.8)	28.7 (24.8–31.5)	.0876

Bold indicates *P* < .05. Ellipses (…) indicate no statistical tests employed.

Abbreviations: Ct, cycle threshold; CT, computed tomography; NA, not available; STB, spinal tuberculosis; TB, tuberculosis; TTP, time to positivity.

^a^Only 15 patients had TB treatment information readily available: 3 patients undergoing open biopsy were receiving treatment for pulmonary TB; 6 of 11 undergoing CT-guided biopsy were receiving antituberculous therapy for pulmonary TB prior to biopsy (length unknown); and 1 of 11 started therapy 2 days prior to biopsy based on spinal magnetic resonance imaging lesions.

^b^TB culture was not performed in 2 patients.

^c^ *rpoB* Ct indicated only for positive samples.

A sensitivity and specificity of 100% were obtained for Xpert Ultra against the composite reference standard based on open biopsy samples, as compared with 89% sensitivity and 100% specificity for CT-guided biopsies (Table 4). When compared with the reference of culture positivity, both biopsy methods had 100% sensitivity; however, CT-guided biopsies had a specificity of 43%, as opposed to 83% for open biopsies, reflecting that a higher number of patients undergoing CT-guided biopsy had initiated treatment prior to the procedure and were more likely to be culture negative (Tables 3 and 4).

**Table 4.** Per-Sample Accuracy of Xpert Ultra for STB Diagnosis Based on Open or CT-Guided Biopsy

	% (No., 95% CI)			
Reference Standard	Sensitivity	Specificity	PPV, %	NPV, %
Open biopsy				
Positive TB				
Composite: culture or histology	100.0 (10/10, 72.2–100.0)	100 (10/10, 72.2–100.0)	100.0	100.0
Culture only	100.0 (8/8, 67.6–100.0)	83.3 (10/12, 55.2–97.0)	80.0	100.0
CT-guided biopsy				
Positive TB				
Composite: culture or histology	89.0 (8/9, 56.5–99.4)	100.0 (2/2, 17.7–100.0)	100.0	67.0
Culture only	100.0 (2/2, 17.7–100.0)	43.0 (3/7,^a^ 15.8–74.9)	33.0	100.0

Abbreviations: CT, computed tomography; NPV, negative predictive value; PPV, positive predictive value; STB, spinal tuberculosis; TB, tuberculosis.

^a^Two had no TB culture performed.

### HIV-1 Status Had No Impact on Culture TTP or Average Xpert Gene Cycles for *Mtb* DNA Detection

There was a significant difference in age between patients with STB who were HIV-1 coinfected and uninfected (*P* = .0089) with older patients being HIV-1 coinfected. We found no difference in *Mtb* load by HIV status, measured by culture TTP (*P* = .6333), Xpert Ultra *rpoB* Ct, or *IS1081-IS6110* Ct (Table 5). No effect of HIV status remained for Xpert Ultra Ct when samples were split into definite and probable STB (Supplementary Table 3).

**Table 5.** Characteristics of Patients With Probable or Definite Spinal TB by HIV-1 Status

	HIV-1, Median (IQR) or No. (%)		
Characteristic	Infected (n = 12)	Uninfected (n = 7)	*P* Value
Age, y	45 (39–51)	29 (21–33)	.**0089**
Male gender	3 (25)	7 (100)	.**0031**
PTB history	8 (67)	1 (14)	.0573
Antituberculous therapy before biopsy	8 (67)	1 (14)	.0573
STB diagnosis			>.9999
Definite	6 (50)	4 (57)	
Probable	6 (50)	3 (43)	
TB culture TTP, d	20 (12–24)	13 (13–15)	.6333
Xpert Ct, cycles			
IS1081-IS6110	18.7 (17.0–21.1)	19.8 (19.3–21.5)	.3283
*rpoB*	26.2 (22.85- 29.45)	26.15 (25.95–30.1)	.5734

*P* values were calculated with a Mann-Whitney test or Fisher exact test. Bold indicates *P* < .05.

Abbreviations: Ct, cycle threshold; PTB, pulmonary tuberculosis; STB, spinal tuberculosis; TB, tuberculosis; TTP, time to positivity.

## DISCUSSION

To our knowledge, this is the first accuracy evaluation of Xpert Ultra for STB diagnosis, comparing 2 biopsy sampling methods against culture and histopathologic diagnosis and assessing the impact of HIV-1 coinfection. We found the following: (1) Xpert Ultra has higher sensitivity than *Mtb* culture to detect *Mtb* in biopsy samples with histopathologic indication of STB; (2) Xpert Ultra had sensitivity ≥89% for open and CT-guided biopsies; and (3) HIV-1 coinfection did not affect *Mtb* detection by Xpert Ultra or culture. Moreover, Xpert Ultra demonstrated superior utility to culture for STB diagnosis in patients who initiated TB treatment for concurrent pulmonary TB. Open biopsies were also more likely to be culture positive than CT-guided biopsies, although CT-guided biopsies were more often obtained from those already undergoing TB treatment for concurrent PTB. Xpert Ultra detected an equivalent amount of *Mtb* DNA in culture-negative and culture-positive samples, irrespective of HIV-1 status.

Previous studies analyzing nonorthopaedic TB samples report high sensitivity and specificity for Xpert Ultra with improved performance over Xpert and culture [12, 15, 18, 20, 22, 35, 36]. A recent meta-analysis also reported a poor overall TB culture yield of 66% (range, 50%–83%) in 299 percutaneous biopsies and confirmed that culture positivity was more likely in open biopsies that collected larger specimens, which may have better represented the site of disease [37].

Results were available much faster with Xpert Ultra than with culture, enabling timely diagnosis and initiation of therapy. Reliance on TB culture may lead to delays in diagnosis and treatment, with resultant serious morbidity. CT-guided biopsy was a necessary alternative to open biopsies during the pandemic, with limited access to operating rooms. Half these samples were culture negative, which underlines the value of the Xpert Ultra test in percutaneous biopsies with limited specimen volume and the characteristic paucibacillary nature of the disease, particularly when patients have already initiated treatment for concurrent PTB. Culture and histopathology are important but time-consuming methods (either by the length of culture or by hands-on time for the technician) and are dependent on good sampling techniques to improve isolation of *Mtb* bacilli. The discrepancy in the success rate of *Mtb* culture vs histopathology in detecting *Mtb* bacilli in patients with STB may be attributed to prior PTB treatment initiation, the paucibacillary nature of STB, or the physiology of *Mtb* at the site of STB, which may render it refractory to growth in normal culture conditions.

Patients with confirmed STB were mostly in their forties. This age peak has been described [5] as being attributed to high STB prevalence in those who are HIV-1 coinfected. We also found that a large portion of patients (60%) had an unsuppressed HIV-1 VL but with reportedly adherent ART. While this could indicate that the ART regimen was inadequate or inadequately taken by the patients, we previously described the potential for the TB-associated inflammatory microenvironment to activate HIV-1 replication in infected cells at the site of TB disease [38]. It remains to be determined whether there is any relationship between blood VL and HIV-1 abundance at the site of STB in those established as receiving ART. For patients with active TB who are receiving ART, the regimen should be further assessed with particular attention to the potential drug-drug interactions between antiretrovirals and TB drugs [39]. Despite this, we confirm that Xpert Ultra performed well in patients with poorly controlled HIV-1 infection.

### Limitations and Future Considerations

Multiple surgeons and radiologists performed the biopsies, which could have led to a heterogeneous sample collection, although methodology reflects current hospital clinical practice. The pandemic affected the health-seeking behavior of patients, leading to a reduced number presenting with STB than previously reported [24].

## CONCLUSIONS

Xpert Ultra had high diagnostic accuracy in open and CT-guided biopsy samples; yet, TB culture was predominantly positive in open biopsy samples and had limited accuracy for CT-guided biopsies, particularly for patients who had initiated PTB treatment at the time of sampling. Xpert Ultra therefore confirmed more STB cases than culture and did so faster. Xpert Ultra on CT-guided biopsy samples is thus an important tool for rapid diagnosis in times of or in hospitals with limited availability to spinal surgery.

## Supplementary Material

ofad621_Supplementary_Data

## Supplementary Data

Supplementary materials are available at *Open Forum Infectious Diseases* online. Consisting of data provided by the authors to benefit the reader, the posted materials are not copyedited and are the sole responsibility of the authors, so questions or comments should be addressed to the corresponding author.

## Notes

***Acknowledgments.*** We acknowledge and thank research nurses Nomsa Yekiso and Nosipho Mncwabe and the surgical and radiologic teams, especially Dr Qonita Hartley, Dr Chad Centner, and Nawaal Adikary.

***Author contributions*.** R. W. conducted data analysis and drafted the manuscript. M. H. and A. K. C. contributed to study design, supported data collection and analysis, and edited the manuscript. N. A. provided intellectual insight and input on diagnostic testing methodology. R. N. D. and M. L. aided study design, input, and physical biopsy sample collection during surgery.

***Disclaimer.*** For the purposes of open access, the authors have applied a CC-BY public copyright to any author-accepted manuscript arising from this submission.

***Financial support*.** This research was funded in whole, or part, by the Wellcome Trust (Grant Number: 203135/Z/16/Z).
