# Supplementary material for: Higher Sensitivity of Xpert MTB/RIF Ultra Over Tuberculosis Culture for the Diagnosis of Spinal Tuberculosis With Open or Computed Tomography–Guided Biopsies
Source: Open Forum Infect Dis. 2023 Dec 7;11(1):ofad621. doi: 10.1093/ofid/ofad621 (PMC10759005; doi:10.1093/ofid/ofad621)
Supplement: ofad621_Supplementary_Data [file ofad621_supplementary_data.docx]

**Higher sensitivity of Xpert MTB/RIF Ultra over TB culture for the diagnosis of spinal tuberculosis using open or CT-guided biopsies.**

Robyn Waters^1,2,3,*^, Maritz Laubscher^1,2^, Robert N Dunn^1,2^, Nawaal Adikary^4^, Anna K Coussens^3,5,6,†^Michael Held^1,2,†^

**Supplementary Materials**

**Table S1.** Histopathological or other diagnosis for the twelve patients not diagnosed with STB.

| **Final diagnosis** | **n (%)** |
| --- | --- |
| Subacute/Chronic osteomyelitis | 3 (26) |
| Metastatic Cell Carcinoma (Cancer) | 1 (8) |
| Mild Acute Inflammation | 1 (8) |
| Lumbar Spine Abscess | 1 (8) |
| Consistent with fracture site | 1 (8) |
| Spondylodiscitis caused by *Escherichia coli* *(E. coli)* | 1 (8) |
| Degenerative changes | 2 (17) |
| Miliary TB | 1 (8) |
| Unknown pathology | 1 (8) |

**Table S2.** **Spinal TB diagnosis by biopsy type stratified by prior treatment initiation.**

|  | **TB Treatment prior to biopsy** | **No TB treatment prior to biopsy** | ***p-value*** |
| --- | --- | --- | --- |
| **Open Biopsy** | | | |
| *Definite STB* | 2 | 6 | >0.9999 |
| *Probable STB* | 1 | 1 |  |
| **CT-guided Biopsy** | | | |
| *Definite STB* | 1 | 1 | >0.9999 |
| *Probable STB* | 5 | 2 |  |
| **CT-guided Biopsy** | | | |
| *Culture positive* | 1 | 1 | 0.4167 |
| *Culture negative* | 6 | 1 |  |

**Table S3.** **TB culture time to positivity and Xpert Ultra gene cycle threshold values for *Definite* and *Probable STB* patients, stratified by HIV status.**

|  | **Definite STB**  **(n=10)** | | | **Probable STB**  **(n=8)^a^** | | |
| --- | --- | --- | --- | --- | --- | --- |
|  | **HIV-1 co-infected**  **(n=6)** | **HIV-1 uninfected**  **(n=4)** | **p-value** | **HIV-1 co-infected** | **HIV-1 uninfected** | **p-value** |
| **Culture TTP, days** | 20 (10-26) | 13 (12-22) | 0.6333 | - | - | - |
| ***IS1081 - IS6110* Ct, cycles** | 16.9 (16.4 – 20.9) | 19.7 (19.1 – 21.6) | 0.3524 | 21.1 (18.3 – 22.8) | 20.7 (18.2 – 24.8) | >0.999 |
| ***rpoB* Ct, cycles** | 22.8 (21.7 – 28.2) | 26.2 (26.1 – 32.3) | 0.2619 | 29.5 (25.3 – 30.9) | 25.9 (22.2 – 31.4) | 0.5714 |

*Values are presented as median (IQR). p-values were calculated using Mann-Whitney tests.*

*TTP, time to positivity. IQR, Interquartile range. rpoB, β subunit of bacterial RNA polymerase. Ct, Cycle Threshold. IS, insertion element. SD, standard deviation.*

*^a^Of the nine probable TB, eight were Xpert Ultra positive, one patient was Xpert Ultra negative but a single acid-fast bacillus was observed on histopathology examination.*
